# RNA 5-Methylcytosine Regulators Contribute to Metabolism Heterogeneity and Predict Prognosis in Ovarian Cancer

**DOI:** 10.3389/fcell.2022.807786

**Published:** 2022-03-18

**Authors:** Jie Xu, Xiaoyi Liu, Yanjie Chen, Yuya Wang, Tao Liu, Ping Yi

**Affiliations:** Department of Obstetrics and Gynecology, The Third Affiliated Hospital of Chongqing Medical University, Chongqing, China

**Keywords:** ovarian cancer, 5-methylcytosine, RNA modification, metabolism heterogeneity, LASSO cox regression

## Abstract

5-Methylcytosine (m^5^C) is an abundant and highly conserved modification in RNAs. The dysregulation of RNA m^5^C methylation has been reported in cancers, but the regulatory network in ovarian cancer of RNA m^5^C methylation-related genes and its implication in metabolic regulation remain largely unexplored. In this study, RNA-sequencing data and clinical information of 374 ovarian cancer patients were downloaded from The Cancer Genome Atlas database, and a total of 14 RNA m^5^C regulators were included. Through unsupervised consensus clustering, two clusters with different m^5^C modification patterns were identified with distinct survivals. According to enrichment analyses, glycosaminoglycan and collagen metabolism–related pathways were specifically activated in cluster 1, whereas fatty acid metabolism–related pathways were enriched in cluster 2, which had better overall survival (OS). Besides the metabolism heterogeneity, the higher sensitivity to platinum and paclitaxel in cluster 2 can further explain the improved OS. Ultimately, a least absolute shrinkage and selection operator prediction model formed by ALYREF, NOP2, and TET2 toward OS was constructed. In conclusion, distinct m^5^C modification pattern exhibited metabolism heterogeneity, different chemotherapy sensitivity, and consequently survival difference, providing evidence for risk stratification.

## Introduction

Ovarian cancer (OVC) is the most lethal gynecological cancer (Siegel et al., 2021). Because of asymptomatic onset and lack of efficient screening tests, more than 75% of patients are diagnosed at an advanced stage with a 5-year survival rate of 29%, in contrast to 92% for early stage (Singer et al., 2003). The standard frontline care is the debulking surgery to no tumor residual and platinum-based adjuvant chemotherapy, with antiangiogenic therapy applied in patients who have suboptimal tumor reduction and stage IV disease (Lheureux et al., 2019). The poly-ADP-ribose polymerase (PARP) inhibitors have been applied in frontline care for maintenance therapy and in patients with recurrence (Kristeleit et al., 2017; Pujade-Lauraine et al., 2017; Moore et al., 2018). However, the moderate activity of PARP inhibitors was found in patients with homologous recombination dysfunction, and a worse therapeutic effect was observed in homologous recombination–proficient patients (Kaufman et al., 2015). Despite initial response to the first-line treatment, 25% of patients have a relapse within 6 months (Jemal et al., 2003), and more than 80% of patients eventually have a recurrence (Kim et al., 2020). Immunotherapy has demonstrated modest response rates of 10% to 15%, despite a large proportion of OVCs with high expression of programmed death ligand 1 (Pujade-Lauraine, 2017). Facing those challenges in diagnosis and treatment, seeking predictive biomarkers could enable early diagnosis, survival prediction, and identification of patient subgroups who would maximally benefit from those treatments (Lheureux et al., 2019). Consequently, investigations are devoted to molecular and function profiling of OVC for optimal biomarkers.

Dysregulation of RNA expression profile is an important hallmark of tumors (Chai et al., 2019a). RNA 5-methylcytosine (m^5^C) modification, the methylation of the fifth carbon in cytosine base in RNA sequences, has emerged as one of the critical posttranscriptional regulators of gene expression and has been identified in tRNA, rRNA, and mRNA (Nombela et al., 2021). The distribution of m^5^C site in mRNA has been reported to be mainly deposited in the coding sequences and enriched around the translation initiation codon (Amort et al., 2017; Yang et al., 2017; Yang et al., 2019; Tang et al., 2020). RNA m^5^C modification is a reversible and dynamic process mediated by a group of proteins named “writers,” “erasers,” and “readers,” which work as methyltransferases (NSUN, DNMT, and TRDMT families), demethylases (TET family), and binding proteins (ALYREF and YBX1), respectively. RNA m^5^C modification has been involved in the regulation of gene expression (Roundtree et al., 2017; Chai et al., 2019b) and thus has participated in a series of physiological and pathological processes including cancers (Chen et al., 2021).

Cancer is considered as a disease characterized by the accumulation of genetic or epigenetic alterations of different oncogenes and tumor suppressors (Nombela et al., 2021). Metabolism reprogramming is another indispensable hallmark of cancer. Mutation of oncogene and tumor suppressors drives the reprogramming of metabolism and rewiring of epigenetic modification. Cancer cell fate can also be modified by epigenetic modification and alteration of metabolites. m^5^C regulator dysregulation has been reported in multiple cancers such as breast cancer, leukemia, bladder cancer, and skin squamous cell carcinoma (Freeman et al., 1991; Blanco et al., 2016; Cheng et al., 2018; Chen et al., 2019). It has also been demonstrated that RNA m^5^C modification could promote glucose metabolism through enhancing PKM2 mRNA stability in bladder cancer (Wang et al., 2021). However, the role of RNA m^5^C regulators-mediated m^5^C methylation modification, as well as its effect on metabolism reprogramming in OVC, remains unclear.

In this study, we revealed the landscape of genetic variation and gene expression of m^5^C regulators in OVC and established a prognostic prediction model formed by ALYRER, NOP2, and TET2 for overall survival (OS). We also dissected the potential roles of m^5^C modification in metabolism heterogeneity and altered chemotherapeutic drug sensitivity, which could result in survival differences of OVC patients.

## Materials and Methods

### Data Resources

The workflow of our study is shown in Supplementary Figure S1A . The fragments per kilobase of exon model per million mapped fragments (FPKM) files of RNA-seq transcriptome data, as well as clinical information of 374 cases of OVC, were downloaded from The Cancer Genome Atlas (TCGA) database. The SOFT formatted matrix files of three Gene Expression Omnibus (GEO) datasets (GSE27651, GSE52037, GSE54388, and GSE19829) (Clough and Barrett, 2016) were downloaded using R package getGEO. The Masked Copy Number Segment data of DNA copy number variation (CNV) data of OVC were downloaded from the Genomic Data Commons (https://portal.gdc.cancer.gov).

### RNA m^5^C Regulators

Fourteen m^5^C regulators including eight writers (NOP2, NSUN2, NSUN3, NSUN4, NSUN5, NSUN6, NSUN7, TRDMT1); four erasers (TET1, TET2, TET3, ALKBH1); and two readers (YBX1, ALYREF) were enrolled in this study (Chen et al., 2021). DMNT3A and DMNT3B were excluded because they have only been reported in *Arabidopsis thaliana* for now.

### m^5^C Regulators Mutation and CNV Analysis

The somatic mutation investigation of m^5^C regulators in pan-cancer and the CNV analysis in OVC were performed using cBioPortal website (www.cbioportal.org) (Gao et al., 2013). The Pan-Cancer Project of TCGA was enrolled for somatic mutation evaluation in pan-cancer. The Pan-Cancer Project of TCGA-OV with both somatic mutation and mRNA data was enrolled for CNV analysis in OVC.

### Differentially Expressed Gene Analysis

Principal component analysis (PCA) using R package FactoMineR and differential gene expression analysis using R package limma (Ritchie et al., 2015) were conducted, in order to display the different profiles of m^5^C regulators between human ovarian surface epithelium (HOSE) and OVC. Differential analysis was also utilized in seeking DEGs that were specifically up-regulated in each cluster. DEGs were defined as genes with *p* < 0.05 and |fold change| >1.2.

### Interaction Between 14 m^5^C Regulators

The protein–protein interaction (PPI) network plot was constructed using the STRING 11.0 b website (https://string-db.org/). The correlation analysis of the m^5^C regulators among mRNA expression and CNV level and between them was conducted by R package corrplot.

### Clustering Analysis of 14 m^5^C Regulators

The ConsensusClusterPlus package (Wilkerson and Hayes, 2010) was performed to identify distinct m^5^C phenotype based on the expression of 14 m^5^C regulators, and 1,000 times repetitions were conducted to ensure the stability of the classification.

### Cluster Function Annotation and Exploration of Cluster Metabolism Heterogeneity

The cluster function annotation was conducted using R package Gene Set Variation Analysis (GSVA) to explore the Gene Ontology (GO) and Kyoto Encyclopedia of Genes and Genomes (KEGG) enrichment among different m^5^C clusters. GO hallmark and KEGG hallmark gene sets were downloaded from MsigDB dataset (http://www.gsea-msigdb.org/gsea/msigdb). Further functional annotation of each m^5^C cluster was performed by R package ClusterProfiler (Yu et al., 2012) using the top 500 expressed genes and DEGs in each cluster for GO and KEGG pathways. Then, Gene Set Enrichment Analysis (GSEA) was performed using cluster DEGs by ClusterProfiler package to further identify up-regulated pathways in the individual cluster. The up-regulated pathways identified in multiple methods were finally visualized in circos plot using R package circlize. Then, metabolic pathways were downloaded from KEGG database including 1,653 human genes assigned to 91 pathways. The GSVA scores of metabolic pathways were calculated using the GSVA package for further correlation with mRNA level of m^5^C regulators.

### Prediction of Drug Sensitivity

The drug sensitivity was predicted using calcPhenotype function in R package oncoPredict (Maeser et al., 2021) based on Genomics of Drug Sensitivity in Cancer cell line dataset (https://www.cancerrxgene.org/). The prediction ability by drug sensitivity score calculation was validated in OVC clinical trial (GSE51373) with area under the curve (AUC) of 0.786. Imputed lower sensitivity score represents higher sensitivity of the drug.

### Cell Culture and Cell Growth

OVCAR3 cells were cultured in Dulbecco modified eagle medium (GIBCO, United States) supplemented with 10% fetal bovine serum (GIBCO, United States), penicillin (GIBCO, United States), and streptomycin (GIBCO, United States) and maintained at 37°C in 5% CO_2_ cell culture incubator. The cells transfected with siRNA targeting TET2 or control (TsingkeBiotechnology, China) were seeded in 96-well plates for the cell viability test. CCK-8 reagent (DOJINDO, Japan) was added into the plate and incubated for 2 h. The cell absorbance at 450-nm wavelengths was measured by using the microplate reader (BioTek, United States) at 0, 1, 2, 3, and 4 days. All experiments were performed in triplicate.

### Western Blot

Cells were collected and lysed with cell lysis buffer (Beyotime, China) on ice for 30 min, and the lysate was obtained by centrifugation at 12,000*g* for 10 min. Proteins were fractionated by sodium dodecyl sulfate–polyacrylamide gel electrophoresis and then transferred onto 0.22-μm NC membranes. The membranes were blocked with 5% nonfat milk in TBS/Tween-20 and blotted with the antibody (anti-TET2; ProteinTech) at 4°C overnight. Corresponding secondary antibodies (ZSGB-BIO, China) were added on the membrane at room temperature for 1.5 h. Immunoreactive bands were visualized using enhanced chemiluminescence detection reagent (Millipore, United States).

### Statistical Analysis

Correlations among different m^5^C regulators were evaluated by Spearman correlation analyses using R package corrplot. Correlations between m^5^C regulators and clinicopathological parameters were evaluated by Spearman correlation analyses using SPSS 25.0. A *χ* ^2^ test was conducted to compare the clinicopathological parameters of clusters. Kruskal–Wallis test was used to compare gene expression among different samples. R packages survival and survminer (Scrucca et al., 2007) were used to perform the univariate Cox proportional hazards analysis and Kaplan–Meier analysis for OS. R package forestplot and survminer were used for visualized the Cox analysis results and survival curves, respectively. Genes with *p* < 0.05 in univariate analysis were selected to the least absolute shrinkage and selection operator (LASSO) method regression analysis using R package glmnet. Patients with survival information were randomly divided into two groups (75% in the training group and 25% in the test group) by createDataPartition function from R package caret. Three gene signature and their corresponding coefficients were determined in the training group by glmnet package, and the risk score was calculated for each patient using the prediction function. The AUC of receiver operating characteristic (ROC) curve was calculated by R package survivalROC (Heagerty and Zheng, 2005). True positive (TP) and false positive (FP) of every patient in the training group were calculated through survivalROC function, and the minimum value of the formula (TP-1)*2 + FP*2 was determined as the best cutoff value. This cutoff value was used in the internal training set, internal testing set, and external testing set to divide the samples into the high-score group and the low-score group. R 4.0.3 was used for all the statistical analyses in this study. *p* < 0.05 is the significance threshold for all the data.

## Results

### Profiles of Genetic Variation and Gene Expression of RNA m^5^C Regulators in Ovarian Cancer

**FIGURE 1:**
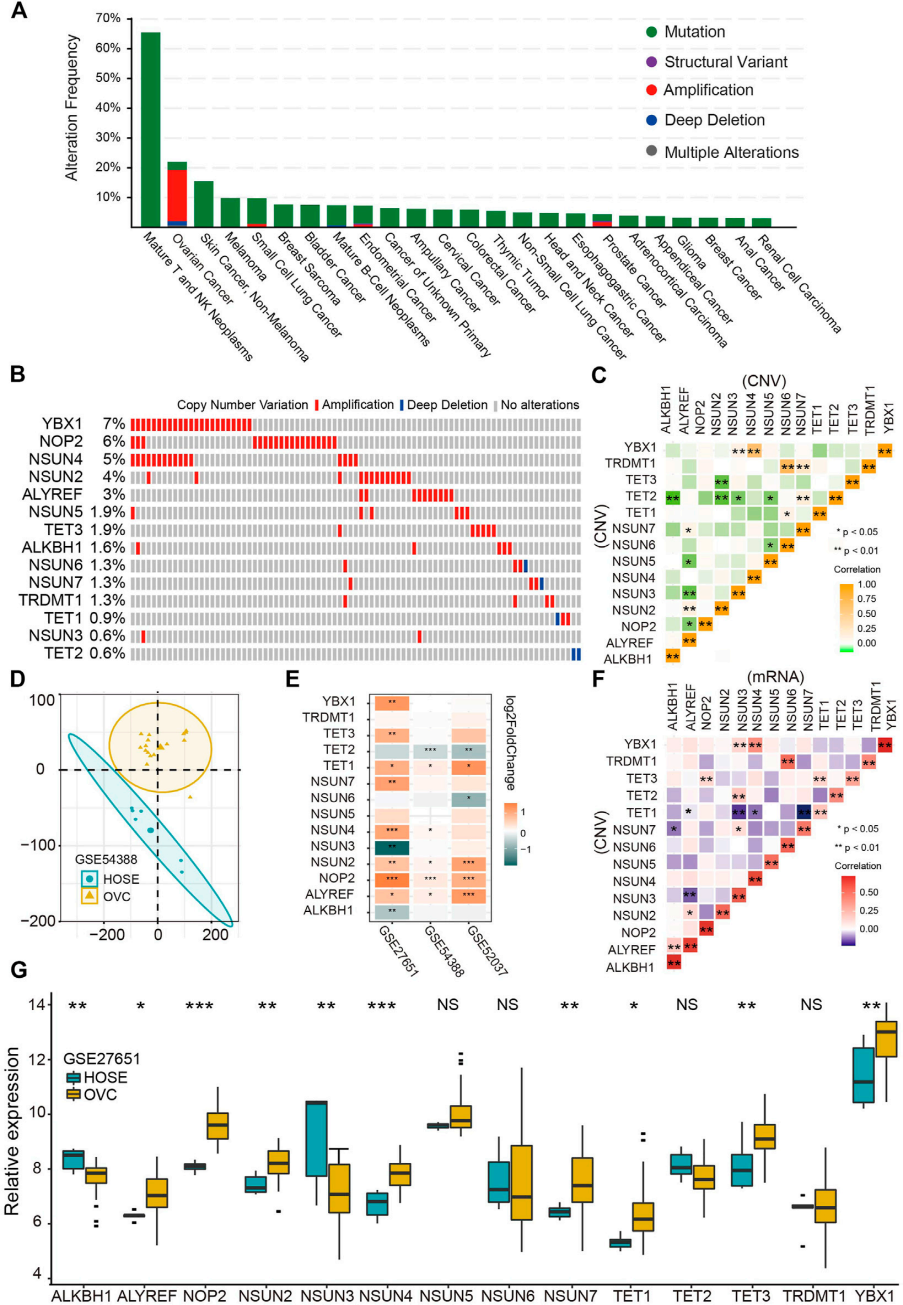
Genetic variation and gene expression of RNA m^5^C regulators in ovarian cancer. **(A)** Genetic alteration frequencies of m^5^C regulators in pan-cancer. **(B)** Copy number variation of m^5^C regulators in ovarian cancer. **(C)** Correlation of copy number variations among m^5^C regulators. **(D)** Principal component analysis for the expression profile of m^5^C regulators to distinguish OVC from HOSE samples in GSE54388 cohort. OVC, ovarian cancer, HOSE, human ovarian surface epithelium. **(E)** The differential expression analysis of 14 m^5^C regulators between OVC and HOSE samples in three independent GEO cohorts. Up-regulated in OVC: orange; up-regulated in HOSE samples: blue. **(F)** Correlation between copy number variation and mRNA level of m^5^C regulators. **(G)** The boxplot of expression of 14 m^5^C regulators in OVC and HOSE samples in GSE27651 cohort.*p < 0.05, **p < 0.01, ***p < 0.001.

First, we comprehensively studied the profile of the genetic variation frequency of m^5^C regulators in the pan-cancer cohort. The amplification is the prevalent variation pattern of m^5^C regulator genes in OVC, and 13 of 14 (92.9%) regulators were amplified (Figures 1A,B). Among those regulators, YBX1, NOP2, and NSUN4 genes exhibited the highest amplification frequencies of 7%, 6%, and 5%, respectively (Figure 1B). The significant positive correlation of CNV among regulators was demonstrated especially between ALYREF and writers, as well as TET2 and writers (Figure 1C). Then, we explored the correlation between CNV and mRNA levels of each regulator and found a significant positive correlation in all 14 regulators (Figure 1D). Differential analysis was further performed to profile the expressions of 14 m^5^C regulators between HOSE and OVC. Consistent with the CNV pattern, most regulators were significantly up-regulated in OVC compared with HOSE tissues, whereas TET2 expression was decreased in two GEO cohorts (Figures 1F,G). Besides, a significant distinction of m^5^C regulators’ expression profiles among HOSE and OVC was illustrated by PCA (Figure 1E).

### The Interaction and Correlation Analysis of RNA m^5^C Regulators in Ovarian Cancer

**FIGURE 2:**
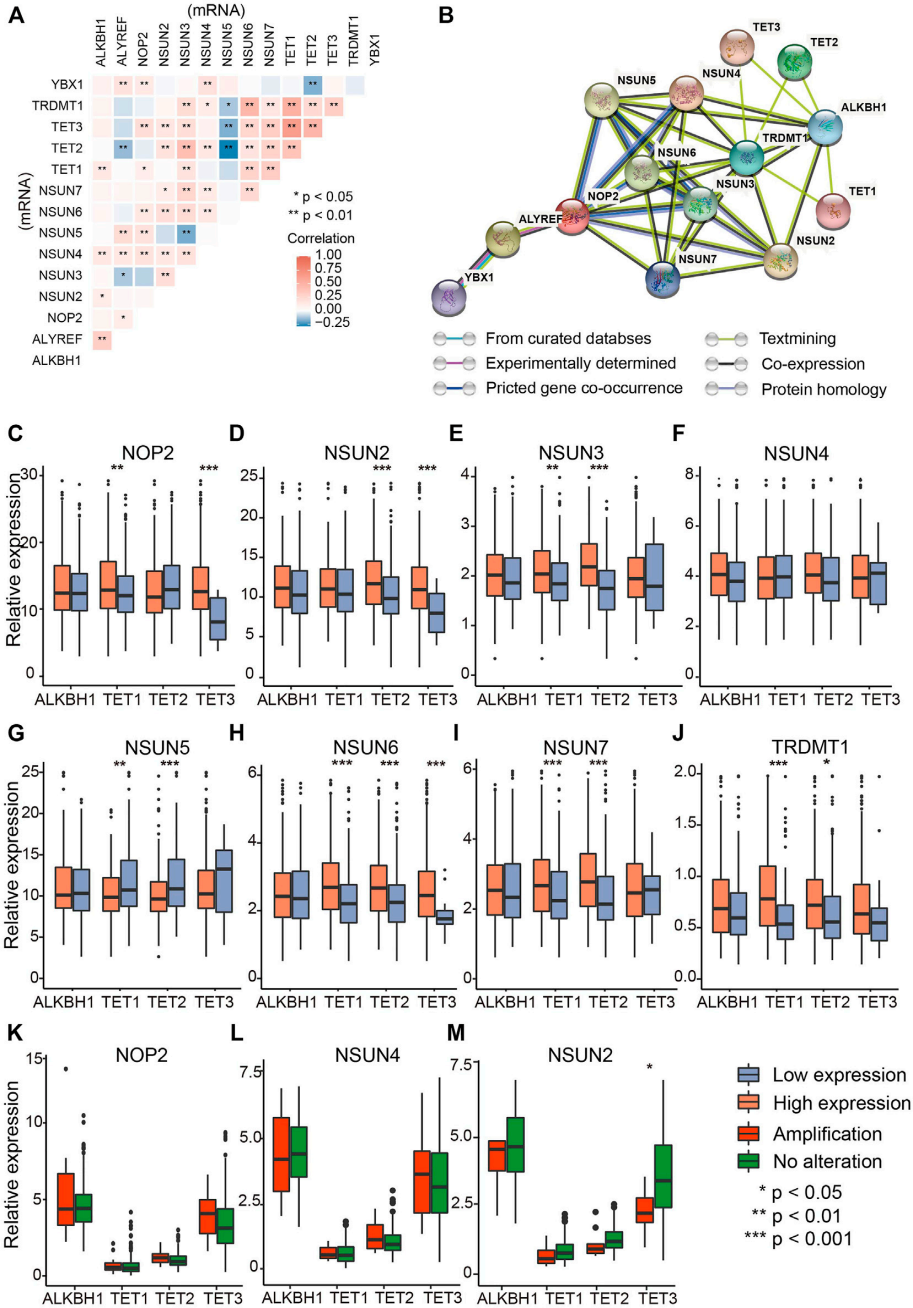
The interaction and correlation analysis between RNA m^5^C regulators in ovarian cancer. **(A)** Correlation of mRNA levels among m^5^C regulators. **(B)** Protein–protein interaction plot among the 14 m^5^C regulators. **(C–J)** Correlations between “writers” (NUSN1, NSUN2, NSUN3, NSUN4, NSUN5, NSUN6, NSUN7, TRDMT1) and “erasers” (TET1, TET2, TET3, ALHBK1) at mRNA level (RPKM). **(K–M)** Differential mRNA levels of “erasers” between amplified type and wild type of “writers” with the highest CNV frequencies (NOP2, NSUN4, and NSUN2).

To testify whether m^5^C regulators have correlations among each other, Spearman correlation analysis of mRNA levels of 14 regulators indicated significant positive correlation among most regulators (Figure 2A). The comprehensive landscape of m^5^C regulators was depicted with the PPI network according to the STRING 11.0 b website (Figure 2B). The writers and erasers had remarkable interactions within each other except the readers (YBX1 and ALYREF). To further investigate the correlations of writers and erasers who work as methyltransferases and demethyltransferases and affect the amount and distribution of m^5^C modification, comparisons of writer gene expression were performed in patients with high and low eraser gene expression (Figures 2C–J) (Zhang et al., 2020). The results showed that writer genes exhibited different correlations with eraser genes. NSUN6 expression was positively correlated with TET1/2/3, whereas NSUN7 expression was negatively correlated with eraser genes TET1/2 (Figures 2H,I). As writer genes NOP2, NSUN4, and NSUN2 have relatively higher amplification (Figure 1B), we analyzed whether the CNV of those writer genes is correlated with eraser genes. Of these, only TET3 was significantly down-regulated in patients with NSUN2 amplification compared with wild type (Figures 2K-M). These data indicate the complex cross-talk among m^5^C regulators in OVC.

### Consensus Clustering of m^5^C RNA Methylation Regulators Identifying Two Clusters With the Distinct OS

**FIGURE 3:**
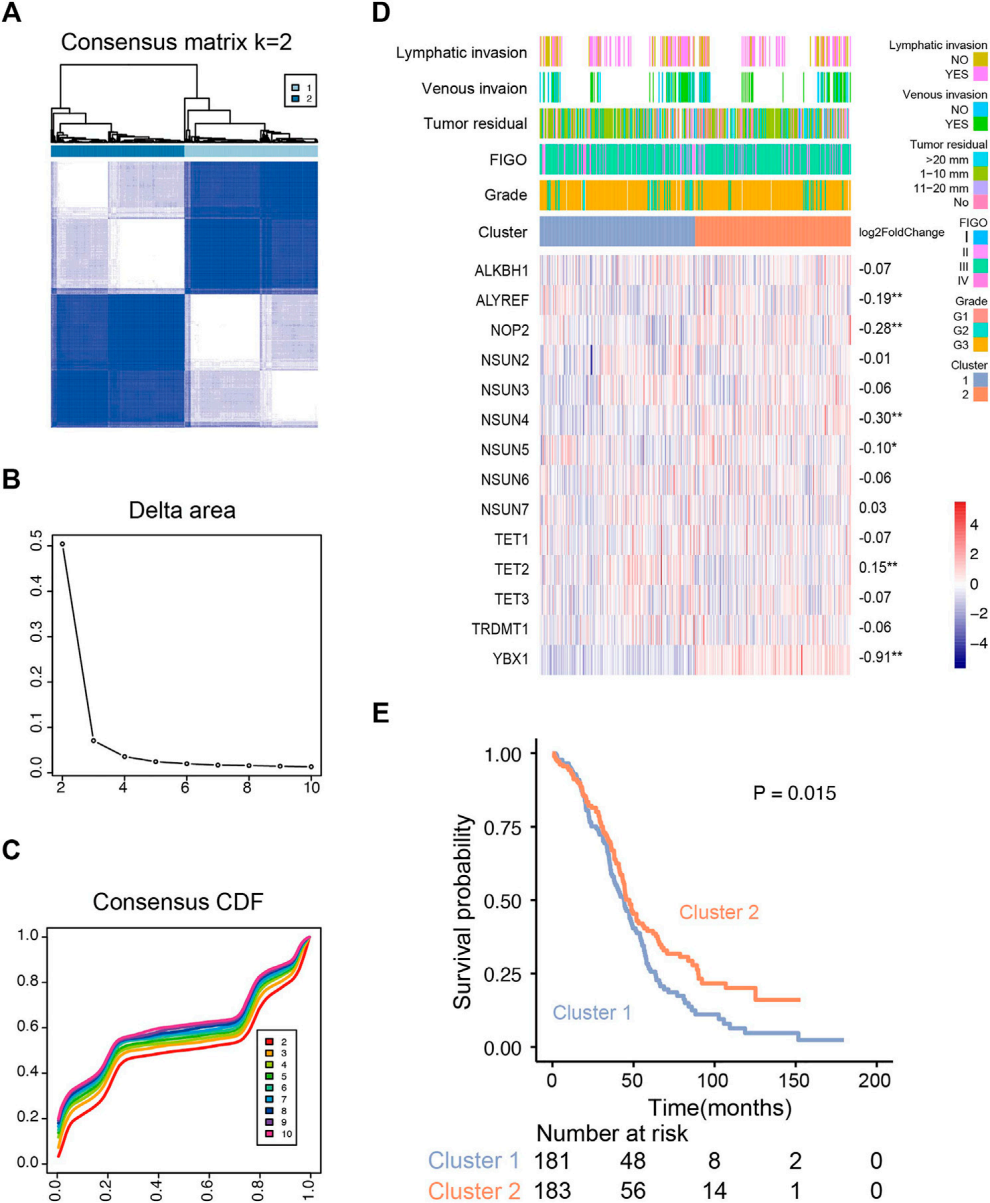
Unsupervised consensus clustering analysis of RNA m^5^C regulators. **(A)** Consensus clustering matrix for the most suitable *k* (k = 2). **(B)** Relative changes in the area under the clustering cumulative function (CDF) curve at k = 2–10. **(C)** Consensus clustering CDF for k = 2–10. **(D)** The m^5^C regulator expression profiles and clinicopathological characteristics in two clusters. **(E)** Kaplan–Meier overall survival curves for ovarian cancer patients of two clusters in the TCGA cohort.

Based on the expression profile of m^5^C regulators in 374 OVC patients (TCGA), we used unsupervised consensus clustering analysis to distinguish different m^5^C modification patterns, and two clusters were identified (Figures 3A–C). k = 2 is the optimal stable clustering when compared with k = 3–8 (Supplementary Figure S1B). Distinct expression profiles of m^5^C RNA methylation regulators, clinicopathological parameters, and log2 (fold change) of regulators are illustrated in Figure 3D. According to *χ* ^2^ test results, no statistical differences were found in lymphatic invasion (*p* = 1), venous invasion (*p* = 0.60), tumor residual (*p* = 0.28), International Federation of Gynecology and Obstetrics stage (*p* = 0.59), and grade (*p* = 0.13) between two clusters. TET2 was significantly up-regulated in cluster 1; ALYREF, NOP2, NSUN4, NSUN5, and YBX1 were substantially up-regulated in cluster 2. Despite similar clinicopathological parameters, the OS of patients in cluster 2 was better than cluster 1 (*p* = 0.015) (Figure 3E). We also examined the correlations of m^5^C regulators and clinicopathological parameters, and the results showed weak correlation between them (Supplementary Figure S2A).

### Functional Annotation Revealing the Metabolism Heterogeneity of the Two Subtypes

**FIGURE 4:**
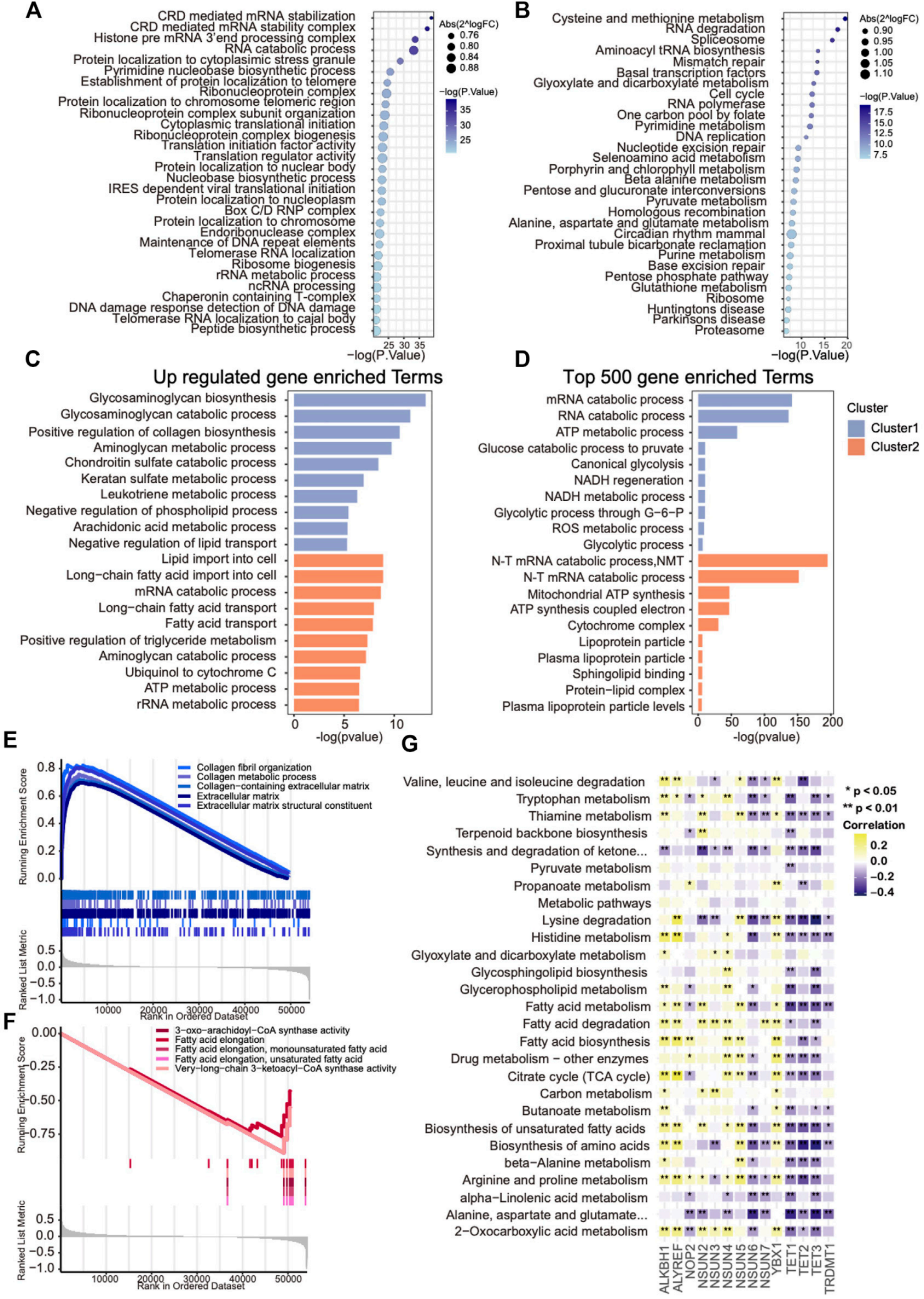
Metabolic heterogeneity in ovarian cancers with distinct m^5^C modification patterns. **(A,B)** Bubble plot showing GSVA enrichment analysis of the top 30 changed GO and KEGG hallmark pathways between two m^5^C clusters. **(C)** The most enriched metabolism-related GO pathways using differentially expressed genes in each m^5^C cluster. **(D)** The most enriched metabolism-related GO pathways using the top 500 up-regulated genes in each m^5^C cluster. **(E,F)** GSEA analysis showing the significant metabolism-related pathways up-regulated in each m^5^C cluster. **(G)** Correlation analysis between GSVA enrichment scores of the changed metabolism-related pathways in each m^5^C cluster and mRNA levels of m^5^C regulators.

To further investigate the activated biological processes that may result in distinct survival in each cluster, functional annotations were conducted through three different methods. First, GSVA scores of GO and KEGG hallmark gene sets were calculated, and the differential analysis was performed to explore the discrepancies in pathways among two clusters, of which metabolism-related pathways occupied more than half of the top 30 enriched pathways (Figures 4A,B). Then, the top 500 expressed genes of each cluster and phenotype-related DEGs were selected for GO enrichment analysis. The results revealed that glycosaminoglycan and collagen metabolism–related pathways were up-regulated in cluster 1, whereas fatty acid metabolism–related pathways were up-regulated in cluster 2, which were further supported by the GSEA results (Figures 4C–F). To further explore the metabolism heterogeneity between the two clusters, the enrichment scores of 91 metabolic pathways downloaded from the KEGG database were calculated, and the varied metabolic pathways between the two clusters were analyzed. Differences were found in 27 of 91 (29.7%) metabolic pathways between two clusters. In addition, a correlation analysis was performed between those metabolic pathway enrichment scores and mRNA levels of m^5^C regulators. A significant negative correlation with these metabolism processes was found in eraser genes and positive correlation in writer genes except for NSUN6 and NOP2 (Figure 4G). Taken together, these results indicate that m^5^C modification might contribute to OVC survival difference through regulating metabolism heterogeneity.

### Representative Metabolism-Related Genes With the Prognostic Value in Different m^5^C Modification Patterns

**FIGURE 5:**
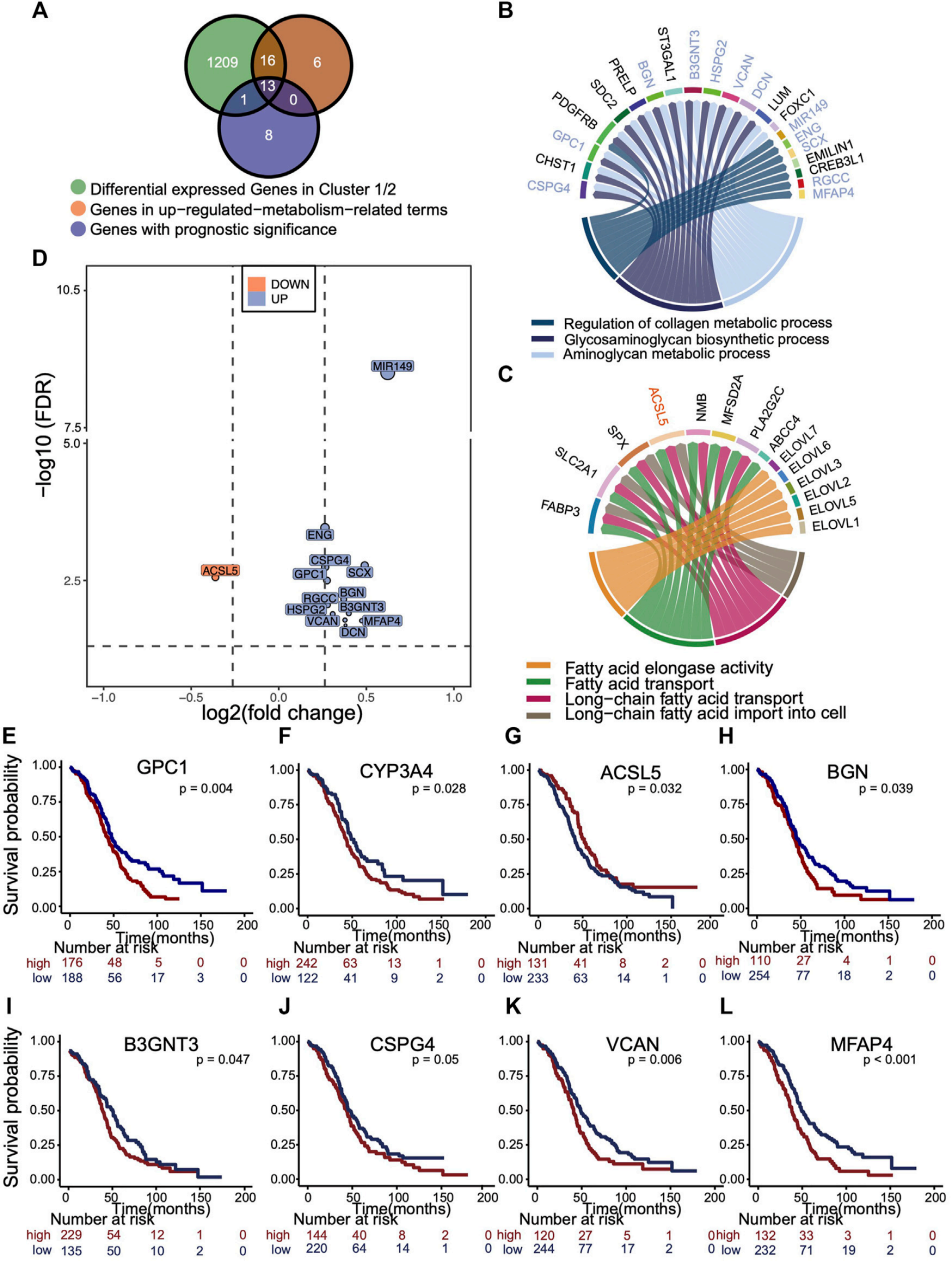
Selection of representative genes differentially expressed in m^5^C cluster, related to metabolism and with prognostic significance. **(A)** Venn plot showing the overlapping analysis of genes that were differentially expressed (fold change >1.5 and *p* < 0.05) in two clusters, enriched in up-regulated metabolic pathways in each cluster, and had the prognostic significance. **(B,C)** *Circus* plot exhibiting the metabolic pathways up-regulated in each m^5^C cluster and those 13 genes with prognostic value were marked. Blue, up-regulated in cluster 1. Orange, up-regulated in cluster 2. **(D)** Volcano plot distributes the relative expressions 13 representative genes with log2 (fold change) and log10 (FDR). The reference is gene expression of cluster 1. Blue, up-regulated in cluster 1. Orange, up-regulated in cluster 2. **(E–L)** Kaplan–Meier overall survival curves of eight representative genes.

An overlapping analysis was made to explore the leading metabolic genes that contributed to survival difference of two clusters (Figure 5A). 13 m^5^C cluster-specific metabolism genes with prognostic values were identified, as is illustrated in the Venn plot. According to the results of GO enrichment analysis using phenotype-related DEGs, GSEA, and GSVA, the collagen, glycosaminoglycan, and aminoglycan metabolism were up-regulated in cluster 1, whereas fatty acid metabolism was up-regulated in cluster 2. We visualized those genes in activated pathways of two m^5^C modification phenotype clusters (Figures 5B,C), and those important genes with prognostic value were marked in the circos plot. The fold change and *p* of 13 representative genes are shown in the volcano plot (Figure 5D). Kaplan–Meier analysis curves for OS in eight representative genes are presented (Figures 5E–L).

### Different Sensitivity to Chemotherapies Between Two Clusters

**FIGURE 6:**
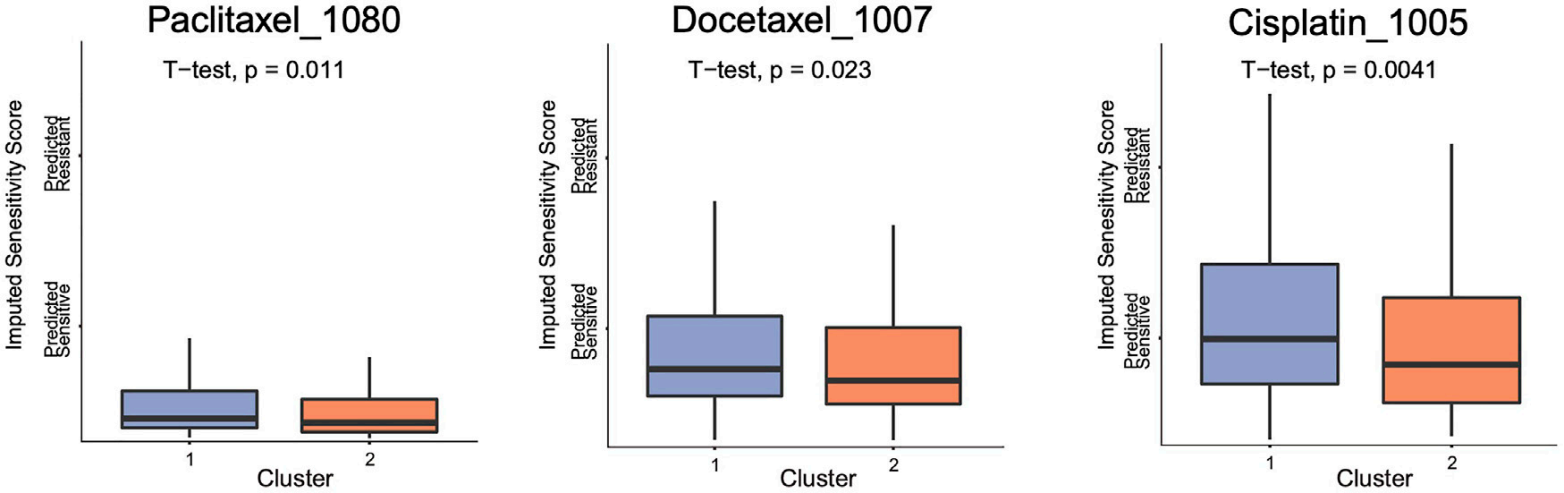
The boxplot of imputed sensitivity score of three chemotherapeutic drugs in two clusters.

In order to detect whether the two clusters with m^5^C modification patterns had different drug sensitivity, we made the sensitivity prediction of 199 drugs for 374 OVC patients from TCGA dataset. Two clusters exhibited different sensitivities in a total of 44 drugs *via t* test for imputed sensitivity score, including Paclitaxel_1,080, Docetaxel_1,007, and Cisplatin_1,005, which were included in the standard chemotherapy of OVC. Higher sensitivities of these three chemotherapeutic drugs were observed in cluster 2 (Figure 6).

### Establishment and Evaluation of a Risk Score Signature With RNA m^5^C Methylation Regulators

**FIGURE 7:**
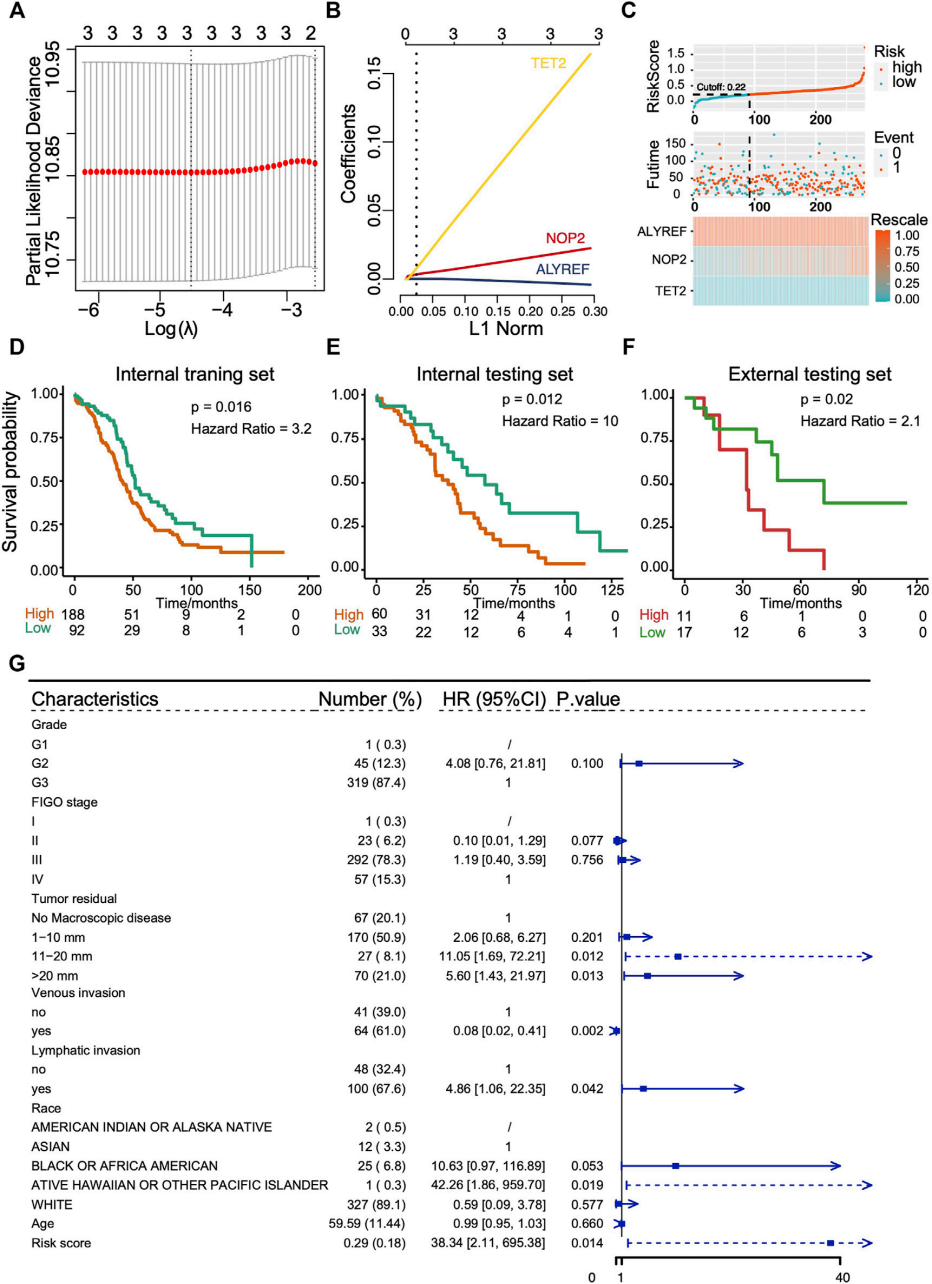
Construction and evaluation of prognostic prediction model with three RNA m^5^C regulators in TCGA cohort and GEO cohort (GSE19829). **(A,B)** LASSO Cox regression analysis results showing the identification of three prognostic risk signature genes, and the risk score model is (ALYREF*-0.00349425170618238) + (NOP2*0.0200758391547147) + (TET2*0.145822477408262). **(C)** The distribution of prognostic signature-based risk score in internal training set. **(D–F)** The Kaplan–Meier overall survival analysis for patients with high score and low score in internal training set, internal testing set, and external testing set. **(G)** The m^5^C risk score in Cox multivariate analysis for OS of ovarian cancer patients.

As clusters with different m^5^C modification patterns exhibited altered metabolism pathways and chemotherapeutic drug sensitivity that were associated with the differences in prognosis, we further explored the predictive value of RNA m^5^C methylation regulators for OS. The univariate survival analyses using Cox proportional hazards models were performed, and three genes with *p* < 0.1 were selected for the LASSO Cox algorithm (Supplementary Figure S2B). Then, the LASSO algorithm with 10 folds of cross-validation was applied to establish a risk score prediction model for OS. Two hundred eighty of 374 OVC patients in TCGA cohorts formed the internal training set, and the rest of 94 patients formed the internal testing set. The GSE19829 formed the external testing set. ALYREF, NOP2, and TET2 finally entered in the model (Figures 7A,B), and the risk core model is (ALYREF*-0.00349425170618238) + (NOP2*0.0200758391547147) + (TET2*0.145822477408262). Patients were then divided into subgroups of low score and high score according to the cutoff value. The distribution of risk scores in the internal training set and internal testing set is demonstrated in Figure 7C and Supplementary Figure S2C, and the results suggest the risk score model could distinguish those patients with poor survivals. Patients with high score exhibit poor OS, which were verified in internal training set (*p* = 0.016, hazard ratio [HR] = 3.2), internal testing set (*p* = 0.012, HR = 10), and external testing set (*p* = 0.02, HR = 2.1) (Figures 7D–F). The AUCs of ROC curves of the prediction were approximately 0.6 (Supplementary Figures S2D,E). To confirm the role of key genes in the risk score signature, *in vitro* experiments were performed. The results showed that knockdown of TET2 in OVC cells could hinder the cell malignant growth, suggesting its potential in the prediction of OS prognosis (Supplementary Figure S2F). Furthermore, multivariate analysis of Cox proportional hazards was performed to further confirm the performance of m^5^C risk score prediction for OS, and the results showed that the m^5^C risk score was an independent prognostic factor (*p* = 0.014, HR = 38.34) (Figure 7G).

## Discussion

In the present study, the metabolism heterogeneity in two clusters based on m^5^C expression profile was annotated through enrichment analyses and found to be significantly correlated with the mRNA levels of m^5^C regulators. Then, 13 metabolism-related DEGs of two m^5^C clusters related RNA m^5^C methylation were identified, which were also associated with OS. Besides metabolism reprogramming, RNA m^5^C regulators could also trigger altered chemotherapeutic drug sensitivity and consequently influence survival in OVC. Ultimately, a prognostic model comprising of ALYREF, NOP2, and TET2 for OS was developed for further verifying predictive value of m^5^C regulators for prognosis in OVC.

As the present diagnosis and screening program in OVC still have limitations, efforts have been made to seek predictive biomarkers related to cancer occurrence and progression. Through these biomarkers, the distinction of subtypes with different prognoses and molecular characteristics will make the identification of patient subgroups who could well respond to a certain treatment or who had worse survival. In the present study, incorporating mRNA data of ALYREF, NOP2, and TET2 could well stratify those patients with worse survivals, providing evidence for clinical practice. The predictive value of m^5^C regulators has also been confirmed in glioma (Wang et al., 2020), breast cancer (Huang et al., 2021), and head and neck squamous cell carcinoma (Xue et al., 2020), and our study made extending support for their predictive role in OVC.

Metabolism reprogramming in OVC has been implicated in the pathogenesis, progression, and target therapy for cancer. In our study, glycosaminoglycan and collagen metabolism–related pathways were activated in cluster 1 and fatty acid metabolism–related pathways were activated in cluster 2. Fatty acids are important components of lipids such as fats, sterol esters, and phospholipids. Lipid metabolism dysregulation has been verified to participate in cancer progression (Li et al., 2021), but it has a more special implication in OVC, as an almost symbiotic relationship exists between OVC and the fat-containing cells in the omentum. FABP4, ELOVL2, and ACSL5 as important genes involved in biosynthesis and transport of fatty acids were found to be up-regulated in cluster 2. FABP4 was highly expressed on the membrane of metastasis OVC cells at the adipocyte–cancer cell interface and mediated lipid accumulation and effect on invasion (Zhao et al., 2019). ELOVL2 has been reported to participate in the biosynthesis of polyunsaturated fatty acids and be involved in tumorigenicity in glioma cancer stem cells (Gimple et al., 2019). ACSL family is responsible for activating long-chain fatty acids, and family members have opposite functions toward carcinogenesis. ACSL5 is nuclear-coded and expressed in the mitochondria and physiologically participates in the proapoptotic sensing of cells acting as a tumor suppressor, which could possibly explain the relatively better prognosis of cluster 2 where ACSL5 was dominantly up-regulated (Quan et al., 2021). The collagen metabolism alteration influences the distribution of collagen and extracellular matrix (ECM) structure, thus affecting cancer progression (Xu et al., 2019). The glycosaminoglycans, another important component of ECM, were found to be involved in multiple signaling pathways related to angiogenesis, cancer invasion, and metastasis (Morla, 2019). VCAN, which is one member of glycosaminoglycan gene sets with leading expression in cluster 1, was previously reported to be up-regulated in ovarian stromal cells and associated with increased microvessel density and poorer survival (Ghosh et al., 2010). In this study, we found the heterogeneity of lipid, glycosaminoglycans, and collagen metabolism in two clusters with distinct m^5^C modification. The stepwise accumulation of altered metabolism at mRNA levels in different m^5^C clusters eventually resulted in distinct prognoses, indicating that the metabolism alteration has prognostic significance.

The role of epigenetic modifications in cancer metabolism reprogramming has been broadly reported, but there is still a lack of disclosure of how RNA m^5^C modification functions in cancer metabolism. It was demonstrated that ALYREF binds the 3′-UTR of PKM2 mRNA and promotes the glucose metabolism of bladder cancer in an m^5^C-dependent manner (Wang et al., 2021). In this study, we found 13 representative metabolic genes that were related to m^5^C RNA methylation in OVC. All of them have positive or negative correlations with RNA m^5^C regulators at the mRNA level (Supplementary Figure S2G). Consequently, experimental verification could be done in the future to verify the regulatory role of RNA m^5^C methylation of those metabolism genes in cancer.

In conclusion, our study depicted the landscape of genetic variation and gene expression of m^5^C regulators in OVC and established a prognostic prediction model formed by ALYRER, NOP2, and TET2 for OS. We also uncovered the indispensable roles of m^5^C modification in metabolism heterogeneity and altered sensitivity to chemotherapeutic drugs.

## Data Availability

The original contributions presented in the study are included in the article/Supplementary Material, further inquiries can be directed to the corresponding author.
