# Supplementary figures and images for: RNA 5-Methylcytosine Regulators Contribute to Metabolism Heterogeneity and Predict Prognosis in Ovarian Cancer
Source: Front Cell Dev Biol. 2022 Mar 18;10:807786. doi: 10.3389/fcell.2022.807786 (PMC8971725; doi:10.3389/fcell.2022.807786)

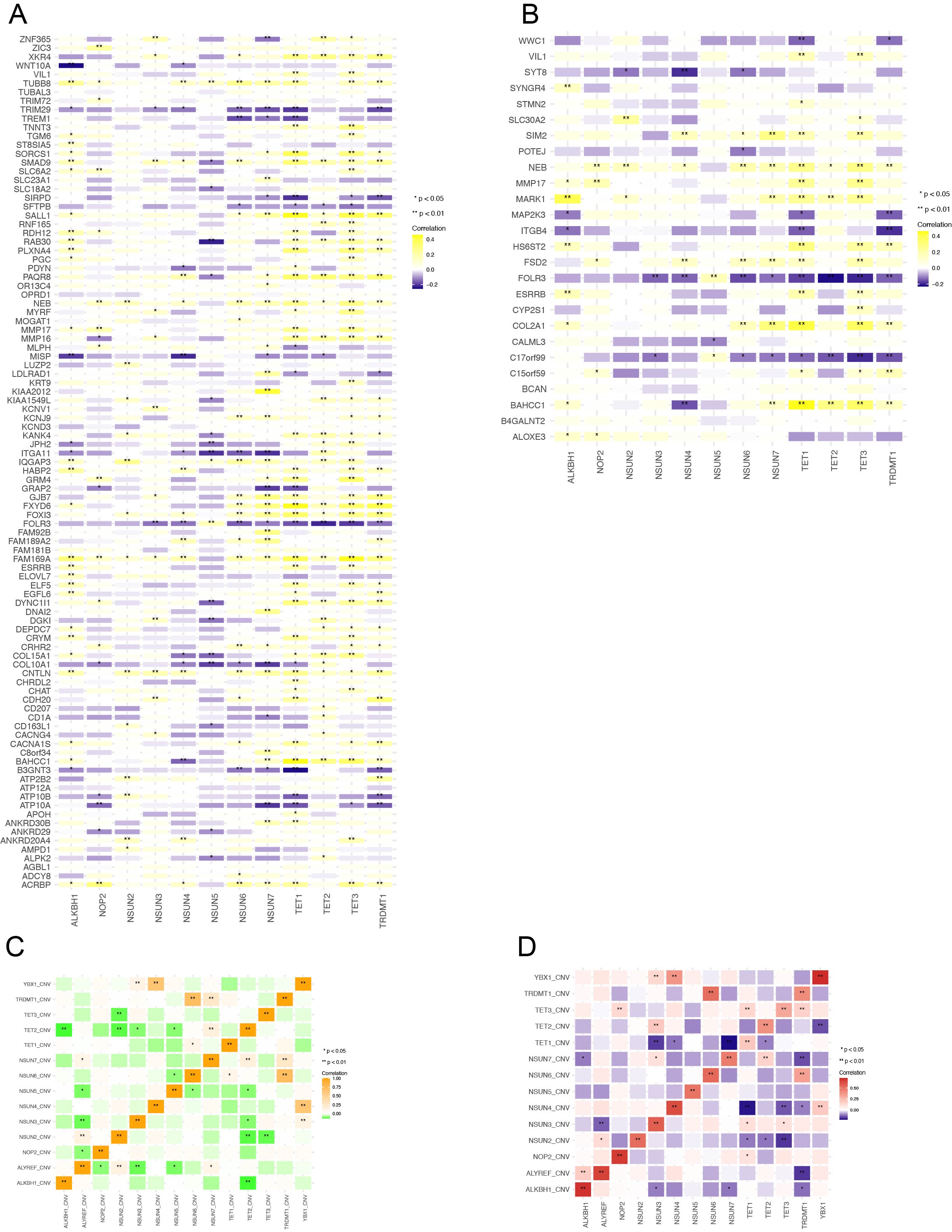

Supplement: Supplementary file 1 [file Image3.JPEG]

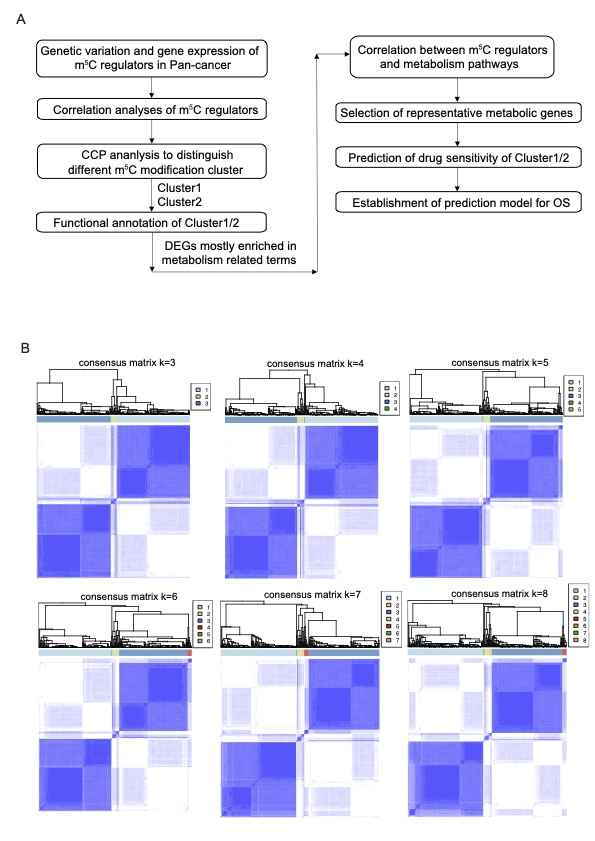

Supplement: Supplementary file 2 [file Image1.JPEG]

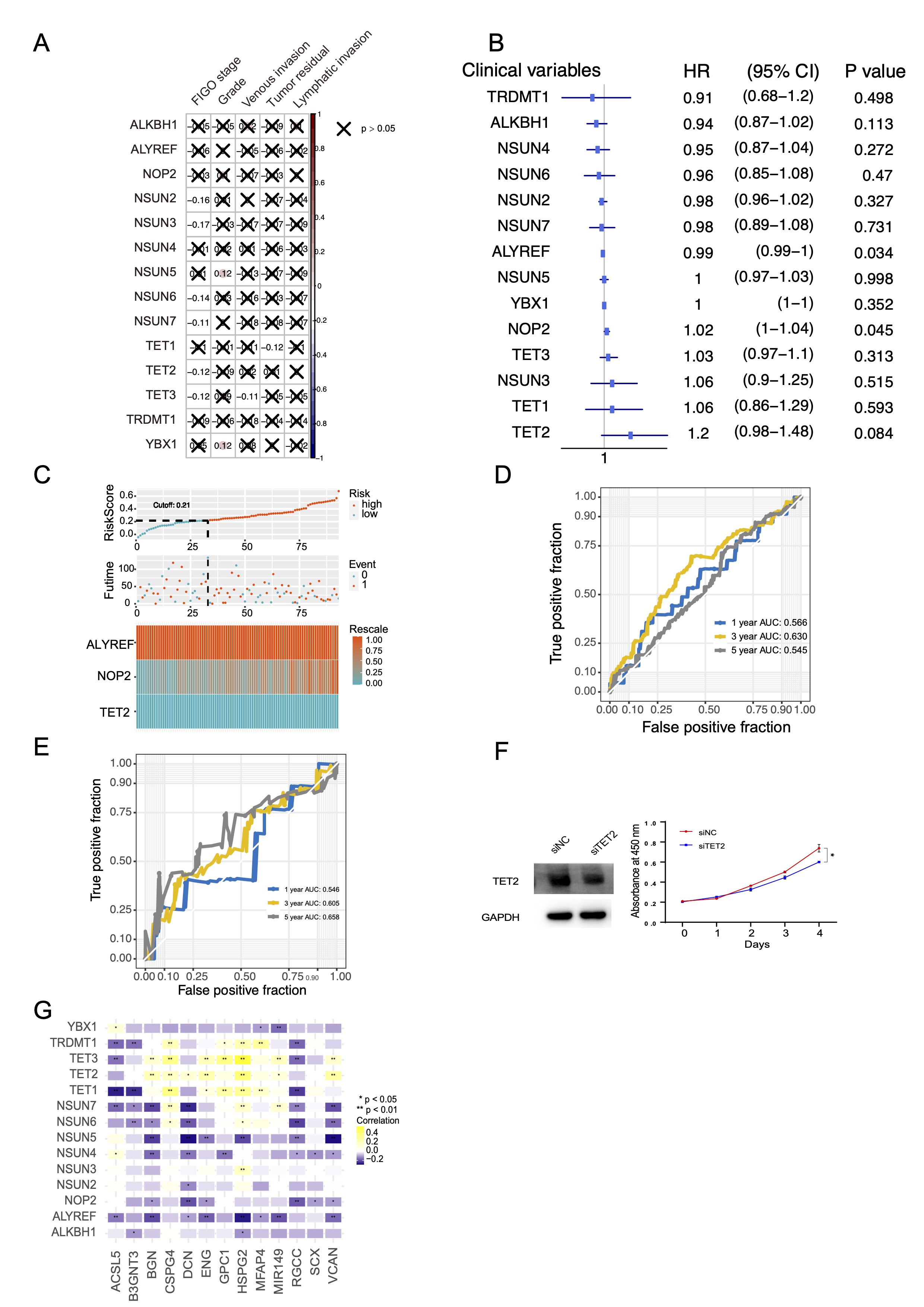

Supplement: Supplementary file 3 [file Image2.JPEG]
